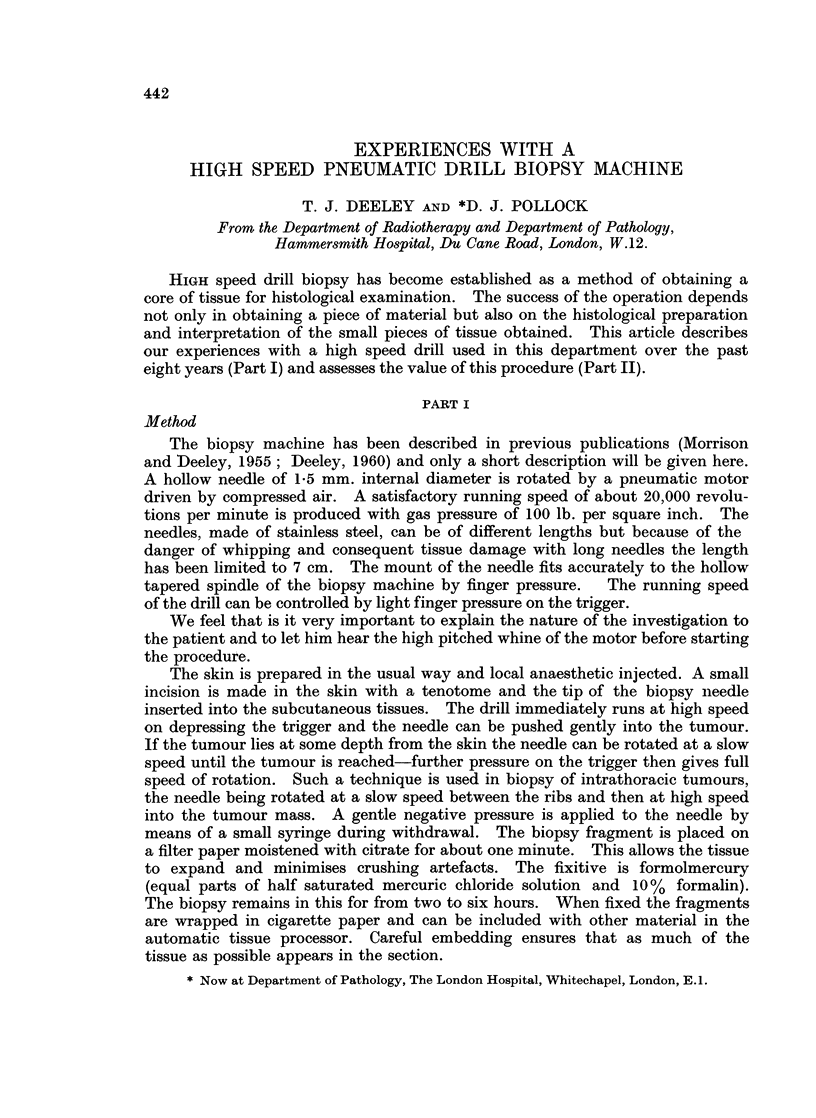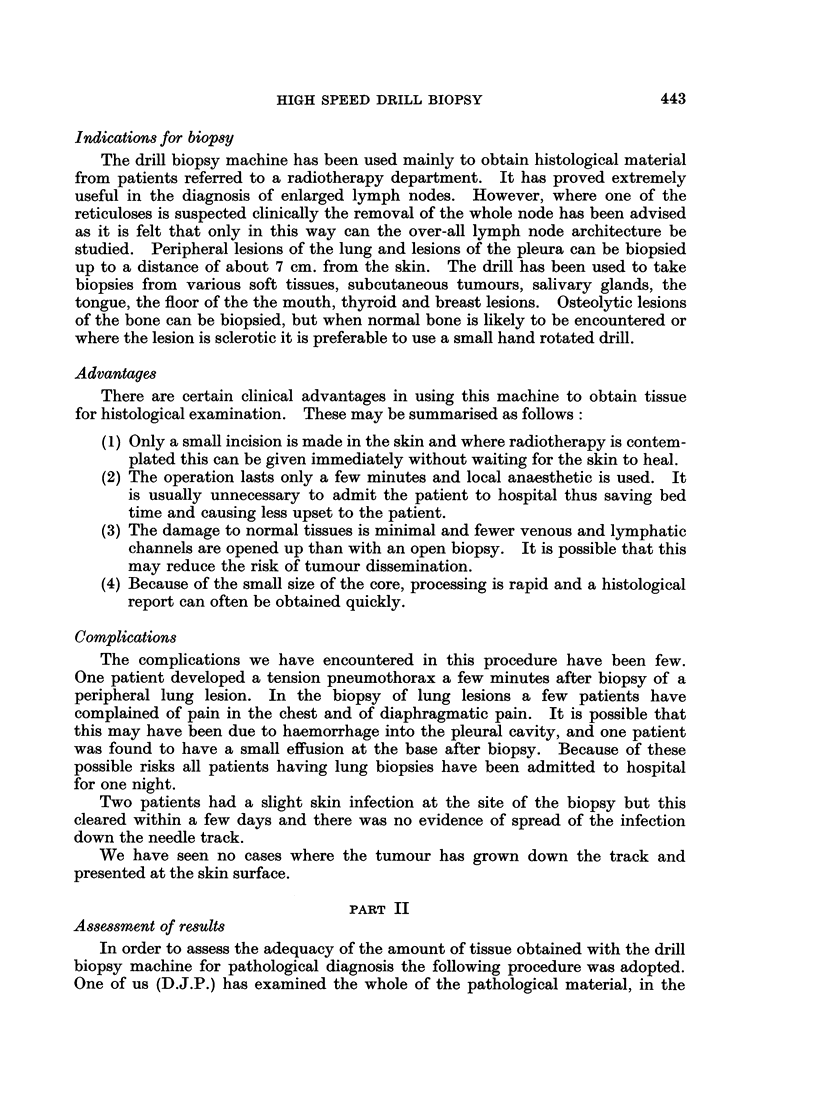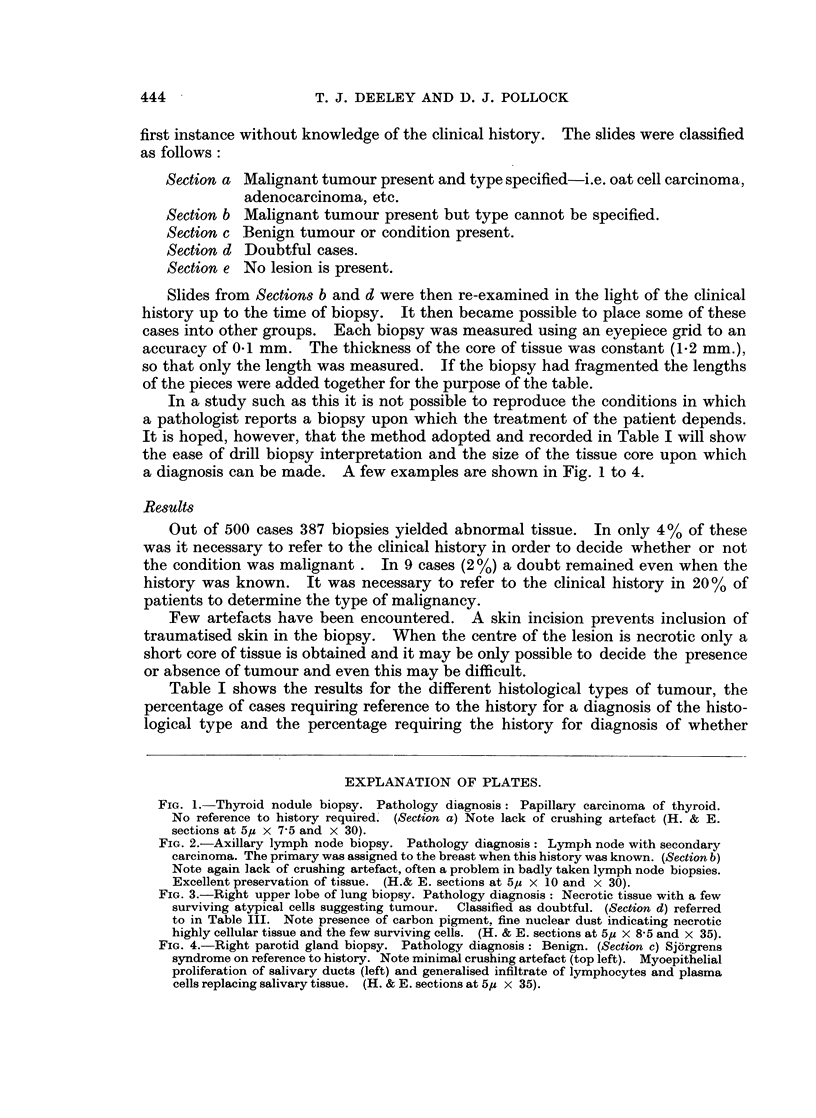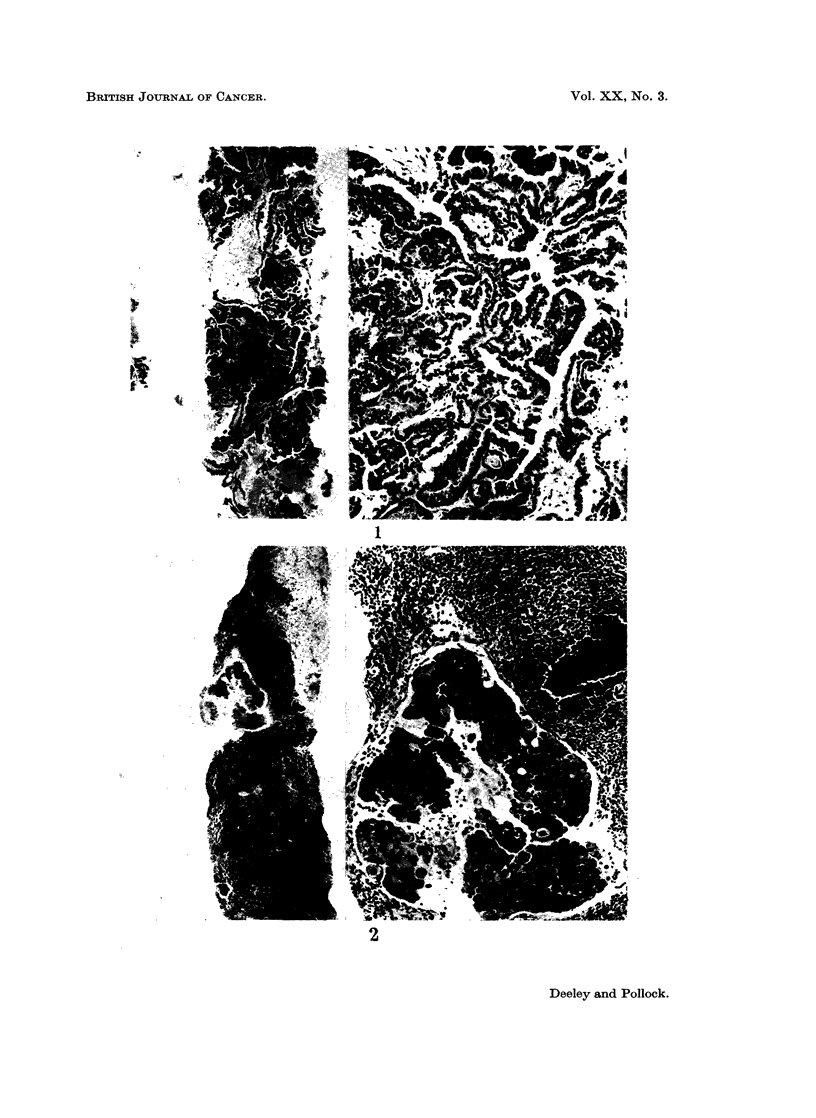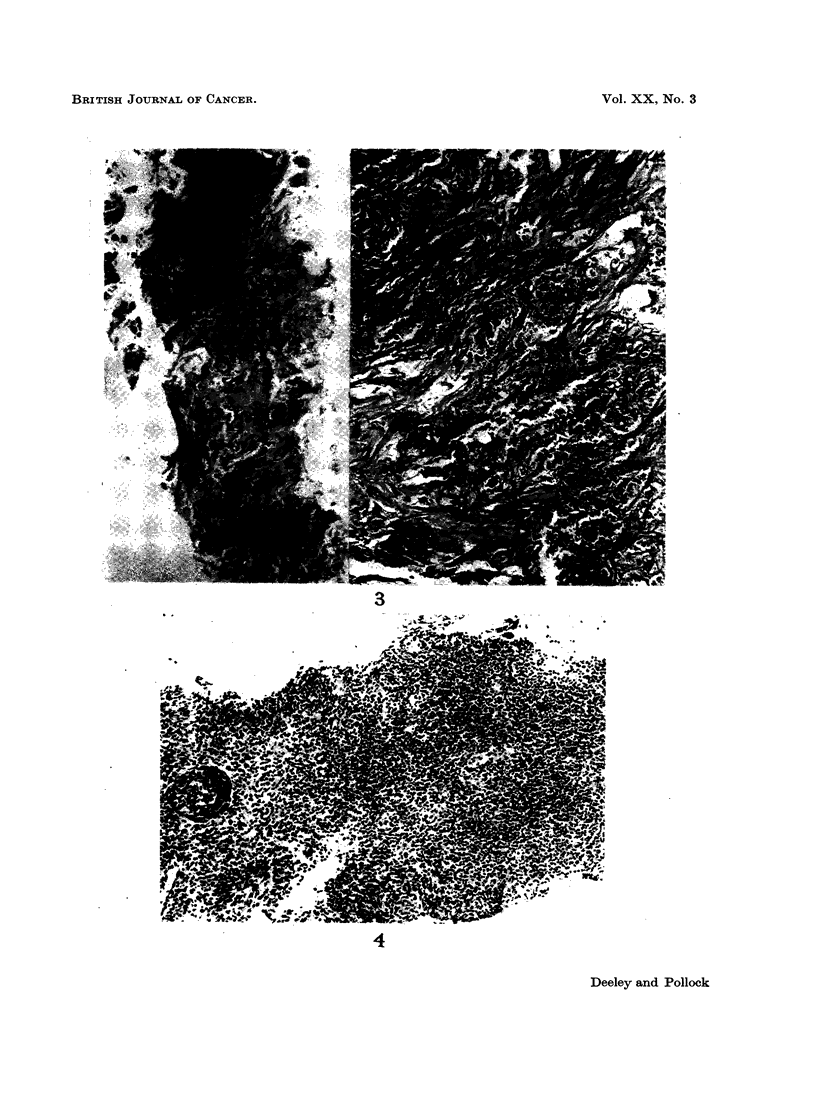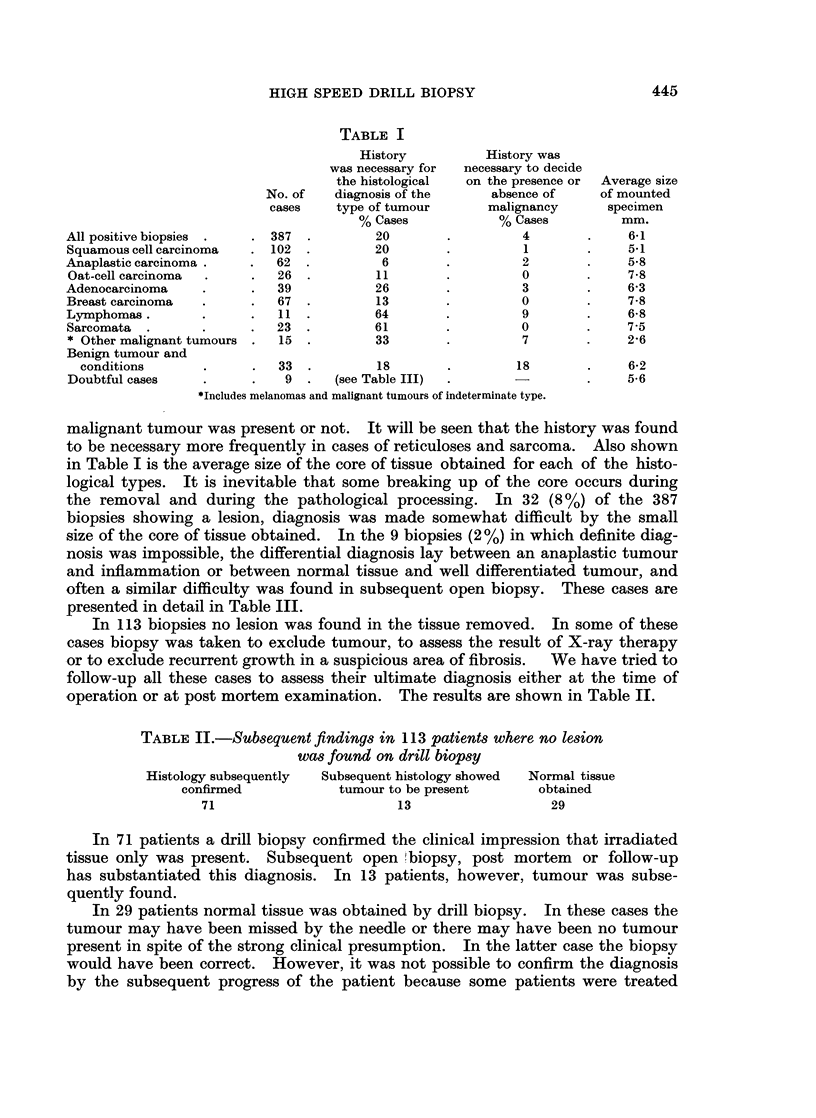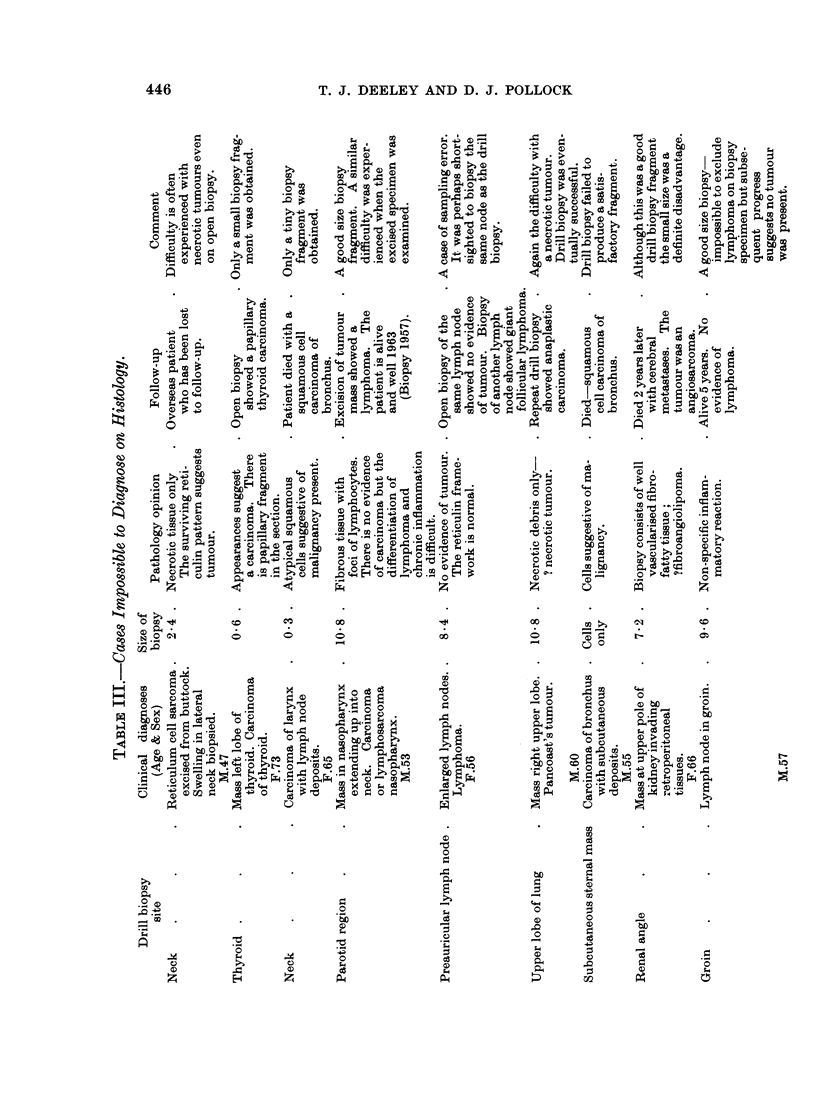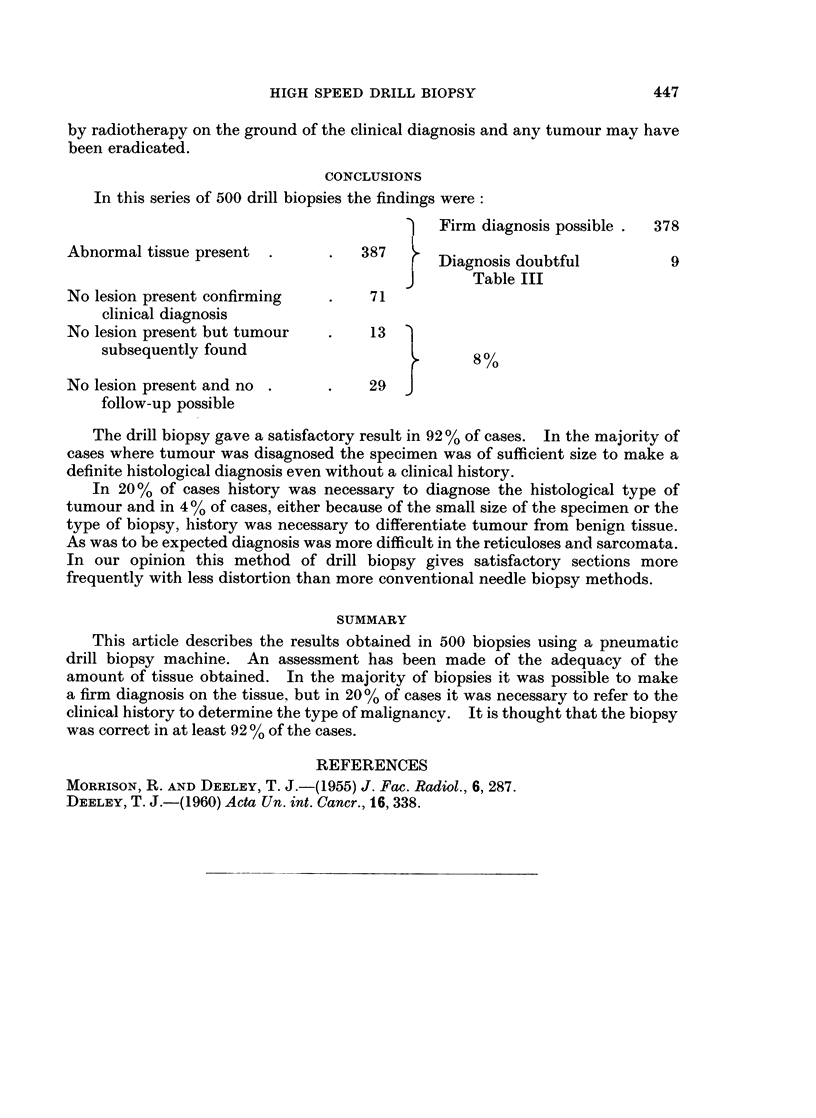# Experiences with a high speed pneumatic drill biopsy machine.

**DOI:** 10.1038/bjc.1966.54

**Published:** 1966-09

**Authors:** T. J. Deeley, D. J. Pollock

## Abstract

**Images:**


### EXPERIENCES WITH A HIGH SPEED PNEUMATIC DRILL BIOPSY MACHINE

T. J. DEELEY AND *D. J. POLLOCK

*From the Department of Radiotherapy and Department of Pathology, Hammersmith Hospital, Du Cane Road, London, W.12.*

HIGH speed drill biopsy has become established as a method of obtaining a core of tissue for histological examination. The success of the operation depends not only in obtaining a piece of material but also on the histological preparation and interpretation of the small pieces of tissue obtained. This article describes our experiences with a high speed drill used in this department over the past eight years (Part I) and assesses the value of this procedure (Part II).

### PART I

### *Method*

The biopsy machine has been described in previous publications (Morrison and Deeley, 1955 ; Deeley, 1960) and only a short description will be given here. A hollow needle of 1·5 mm. internal diameter is rotated by a pneumatic motor driven by compressed air. A satisfactory running speed of about 20,000 revolutions per minute is produced with gas pressure of 100 lb. per square inch. The needles, made of stainless steel, can be of different lengths but because of the danger of whipping and consequent tissue damage with long needles the length has been limited to 7 cm. The mount of the needle fits accurately to the hollow tapered spindle of the biopsy machine by finger pressure. The running speed of the drill can be controlled by light finger pressure on the trigger.

We feel that is it very important to explain the nature of the investigation to the patient and to let him hear the high pitched whine of the motor before starting the procedure.

The skin is prepared in the usual way and local anaesthetic injected. A small incision is made in the skin with a tenotome and the tip of the biopsy needle inserted into the subcutaneous tissues. The drill immediately runs at high speed on depressing the trigger and the needle can be pushed gently into the tumour. If the tumour lies at some depth from the skin the needle can be rotated at a slow speed until the tumour is reached—further pressure on the trigger then gives full speed of rotation. Such a technique is used in biopsy of intrathoracic tumours, the needle being rotated at a slow speed between the ribs and then at high speed into the tumour mass. A gentle negative pressure is applied to the needle by means of a small syringe during withdrawal. The biopsy fragment is placed on a filter paper moistened with citrate for about one minute. This allows the tissue to expand and minimises crushing artefacts. The fixitive is formolmercury (equal parts of half saturated mercuric chloride solution and 10% formalin). The biopsy remains in this for from two to six hours. When fixed the fragments are wrapped in cigarette paper and can be included with other material in the automatic tissue processor. Careful embedding ensures that as much of the tissue as possible appears in the section.

* Now at Department of Pathology, The London Hospital, Whitechapel, London, E.1.

*Indications for biopsy*

The drill biopsy machine has been used mainly to obtain histological material from patients referred to a radiotherapy department. It has proved extremely useful in the diagnosis of enlarged lymph nodes. However, where one of the reticuloses is suspected clinically the removal of the whole node has been advised as it is felt that only in this way can the over-all lymph node architecture be studied. Peripheral lesions of the lung and lesions of the pleura can be biopsied up to a distance of about 7 cm. from the skin. The drill has been used to take biopsies from various soft tissues, subcutaneous tumours, salivary glands, the tongue, the floor of the the mouth, thyroid and breast lesions. Osteolytic lesions of the bone can be biopsied, but when normal bone is likely to be encountered or where the lesion is sclerotic it is preferable to use a small hand rotated drill.

*Advantages*

There are certain clinical advantages in using this machine to obtain tissue for histological examination. These may be summarised as follows :

(1) Only a small incision is made in the skin and where radiotherapy is contemplated this can be given immediately without waiting for the skin to heal.
(2) The operation lasts only a few minutes and local anaesthetic is used. It is usually unnecessary to admit the patient to hospital thus saving bed time and causing less upset to the patient.
(3) The damage to normal tissues is minimal and fewer venous and lymphatic channels are opened up than with an open biopsy. It is possible that this may reduce the risk of tumour dissemination.
(4) Because of the small size of the core, processing is rapid and a histological report can often be obtained quickly.

*Complications*

The complications we have encountered in this procedure have been few. One patient developed a tension pneumothorax a few minutes after biopsy of a peripheral lung lesion. In the biopsy of lung lesions a few patients have complained of pain in the chest and of diaphragmatic pain. It is possible that this may have been due to haemorrhage into the pleural cavity, and one patient was found to have a small effusion at the base after biopsy. Because of these possible risks all patients having lung biopsies have been admitted to hospital for one night.

Two patients had a slight skin infection at the site of the biopsy but this cleared within a few days and there was no evidence of spread of the infection down the needle track.

We have seen no cases where the tumour has grown down the track and presented at the skin surface.

### PART II

*Assessment of results*

In order to assess the adequacy of the amount of tissue obtained with the drill biopsy machine for pathological diagnosis the following procedure was adopted. One of us (D.J.P.) has examined the whole of the pathological material, in the first instance without knowledge of the clinical history. The slides were classified as follows :

*Section a* Malignant tumour present and type specified—i.e. oat cell carcinoma, adenocarcinoma, etc.
*Section b* Malignant tumour present but type cannot be specified.
*Section c* Benign tumour or condition present.
*Section d* Doubtful cases.
*Section e* No lesion is present.

Slides from *Sections b* and *d* were then re-examined in the light of the clinical history up to the time of biopsy. It then became possible to place some of these cases into other groups. Each biopsy was measured using an eyepiece grid to an accuracy of 0·1 mm. The thickness of the core of tissue was constant (1·2 mm.), so that only the length was measured. If the biopsy had fragmented the lengths of the pieces were added together for the purpose of the table.

In a study such as this it is not possible to reproduce the conditions in which a pathologist reports a biopsy upon which the treatment of the patient depends. It is hoped, however, that the method adopted and recorded in Table I will show the ease of drill biopsy interpretation and the size of the tissue core upon which a diagnosis can be made. A few examples are shown in Fig. 1 to 4.

### *Results*

Out of 500 cases 387 biopsies yielded abnormal tissue. In only 4% of these was it necessary to refer to the clinical history in order to decide whether or not the condition was malignant . In 9 cases (2%) a doubt remained even when the history was known. It was necessary to refer to the clinical history in 20% of patients to determine the type of malignancy.

Few artefacts have been encountered. A skin incision prevents inclusion of traumatised skin in the biopsy. When the centre of the lesion is necrotic only a short core of tissue is obtained and it may be only possible to decide the presence or absence of tumour and even this may be difficult.

Table I shows the results for the different histological types of tumour, the percentage of cases requiring reference to the history for a diagnosis of the histological type and the percentage requiring the history for diagnosis of whether

---

EXPLANATION OF PLATES.

FIG. 1.—Thyroid nodule biopsy. Pathology diagnosis : Papillary carcinoma of thyroid. No reference to history required. (*Section a*) Note lack of crushing artefact (H. & E. sections at 5$\mu$ × 7·5 and × 30).

FIG. 2.—Axillary lymph node biopsy. Pathology diagnosis : Lymph node with secondary carcinoma. The primary was assigned to the breast when this history was known. (*Section b*) Note again lack of crushing artefact, often a problem in badly taken lymph node biopsies. Excellent preservation of tissue. (H.& E. sections at 5$\mu$ × 10 and × 30).

FIG. 3.—Right upper lobe of lung biopsy. Pathology diagnosis : Necrotic tissue with a few surviving atypical cells suggesting tumour. Classified as doubtful. (*Section d*) referred to in Table III. Note presence of carbon pigment, fine nuclear dust indicating necrotic highly cellular tissue and the few surviving cells. (H. & E. sections at 5$\mu$ × 8·5 and × 35).

FIG. 4.—Right parotid gland biopsy. Pathology diagnosis : Benign. (*Section c*) Sjörgrens syndrome on reference to history. Note minimal crushing artefact (top left). Myoepithelial proliferation of salivary ducts (left) and generalised infiltrate of lymphocytes and plasma cells replacing salivary tissue. (H. & E. sections at 5$\mu$ × 35).

1

2

3

4

TABLE I

| | No. of cases | History was necessary for the histological diagnosis of the type of tumour % Cases | History was necessary to decide on the presence or absence of malignancy % Cases | Average size of mounted specimen mm. |
|---|---|---|---|---|
| All positive biopsies | 387 | 20 | 4 | 6·1 |
| Squamous cell carcinoma | 102 | 20 | 1 | 5·1 |
| Anaplastic carcinoma | 62 | 6 | 2 | 5·8 |
| Oat-cell carcinoma | 26 | 11 | 0 | 7·8 |
| Adenocarcinoma | 39 | 26 | 3 | 6·3 |
| Breast carcinoma | 67 | 13 | 0 | 7·8 |
| Lymphomas | 11 | 64 | 9 | 6·8 |
| Sarcomata | 23 | 61 | 0 | 7·5 |
| * Other malignant tumours | 15 | 33 | 7 | 2·6 |
| Benign tumour and conditions | 33 | 18 | 18 | 6·2 |
| Doubtful cases | 9 | (see Table III) | — | 5·6 |

*Includes melanomas and malignant tumours of indeterminate type.

malignant tumour was present or not. It will be seen that the history was found to be necessary more frequently in cases of reticuloses and sarcoma. Also shown in Table I is the average size of the core of tissue obtained for each of the histological types. It is inevitable that some breaking up of the core occurs during the removal and during the pathological processing. In 32 (8%) of the 387 biopsies showing a lesion, diagnosis was made somewhat difficult by the small size of the core of tissue obtained. In the 9 biopsies (2%) in which definite diagnosis was impossible, the differential diagnosis lay between an anaplastic tumour and inflammation or between normal tissue and well differentiated tumour, and often a similar difficulty was found in subsequent open biopsy. These cases are presented in detail in Table III.

In 113 biopsies no lesion was found in the tissue removed. In some of these cases biopsy was taken to exclude tumour, to assess the result of X-ray therapy or to exclude recurrent growth in a suspicious area of fibrosis. We have tried to follow-up all these cases to assess their ultimate diagnosis either at the time of operation or at post mortem examination. The results are shown in Table II.

TABLE II.—*Subsequent findings in* 113 *patients where no lesion was found on drill biopsy*

| Histology subsequently confirmed | Subsequent histology showed tumour to be present | Normal tissue obtained |
|---|---|---|
| 71 | 13 | 29 |

In 71 patients a drill biopsy confirmed the clinical impression that irradiated tissue only was present. Subsequent open biopsy, post mortem or follow-up has substantiated this diagnosis. In 13 patients, however, tumour was subsequently found.

In 29 patients normal tissue was obtained by drill biopsy. In these cases the tumour may have been missed by the needle or there may have been no tumour present in spite of the strong clinical presumption. In the latter case the biopsy would have been correct. However, it was not possible to confirm the diagnosis by the subsequent progress of the patient because some patients were treated

TABLE III.—*Cases Impossible to Diagnose on Histology.*

| Drill biopsy site | Clinical diagnoses (Age & Sex) | Size of biopsy | Pathology opinion | Follow-up | Comment |
|---|---|---|---|---|---|
| Neck | Reticulum cell sarcoma excised from buttock. Swelling in lateral neck biopsied. M.47 | 2·4 | Necrotic tissue only The surviving reticulin pattern suggests tumour. | Overseas patient who has been lost to follow-up. | Difficulty is often experienced with necrotic tumours even on open biopsy. |
| Thyroid | Mass left lobe of thyroid. Carcinoma of thyroid. F.73 | 0·6 | Appearances suggest a carcinoma. There is papillary fragment in the section. | Open biopsy showed a papillary thyroid carcinoma. | Only a small biopsy fragment was obtained. |
| Neck | Carcinoma of larynx with lymph node deposits. F.65 | 0·3 | Atypical squamous cells suggestive of malignancy present. | Patient died with a squamous cell carcinoma of bronchus. | Only a tiny biopsy fragment was obtained. |
| Parotid region | Mass in nasopharynx extending up into neck. Carcinoma or lymphosarcoma nasopharynx. M.53 | 10·8 | Fibrous tissue with foci of lymphocytes. There is no evidence of carcinoma but the differentiation of lymphoma and chronic inflammation is difficult. | Excision of tumour mass showed a lymphoma. The patient is alive and well 1963 (Biopsy 1957). | A good size biopsy fragment. A similar difficulty was experienced when the excised specimen was examined. |
| Preauricular lymph node | Enlarged lymph nodes. Lymphoma. F.56 | 8·4 | No evidence of tumour. The reticulin framework is normal. | Open biopsy of the same lymph node showed no evidence of tumour. Biopsy of another lymph node showed giant follicular lymphoma. | A case of sampling error. It was perhaps short-sighted to biopsy the same node as the drill biopsy. |
| Upper lobe of lung | Mass right upper lobe. Pancoast's tumour. M.60 | 10·8 | Necrotic debris only—? necrotic tumour. | Repeat drill biopsy showed anaplastic carcinoma. | Again the difficulty with a necrotic tumour. Drill biopsy was eventually successful. |
| Subcutaneous sternal mass | Carcinoma of bronchus with subcutaneous deposits. M.55 | Cells only | Cells suggestive of malignancy. | Died—squamous cell carcinoma of bronchus. | Drill biopsy failed to produce a satisfactory fragment. |
| Renal angle | Mass at upper pole of kidney invading retroperitoneal tissues. F.66 | 7·2 | Biopsy consists of well vascularised fibro-fatty tissue ; ?fibroangiolipoma. | Died 2 years later with cerebral metastases. The tumour was an angiosarcoma. | Although this was a good drill biopsy fragment the small size was a definite disadvantage. |
| Groin | Lymph node in groin. M.57 | 9·6 | Non-specific inflammatory reaction. | Alive 5 years. No evidence of lymphoma. | A good size biopsy—impossible to exclude lymphoma on biopsy specimen but subsequent progress suggests no tumour was present. |

by radiotherapy on the ground of the clinical diagnosis and any tumour may have been eradicated.

### CONCLUSIONS

In this series of 500 drill biopsies the findings were :

| | | | |
|---|---|---|---|
| Abnormal tissue present . . | 387 | Firm diagnosis possible . | 378 |
| | | Diagnosis doubtful Table III | 9 |
| No lesion present confirming clinical diagnosis . | 71 | | |
| No lesion present but tumour subsequently found . | 13 | 8% | |
| No lesion present and no follow-up possible . . | 29 | | |

The drill biopsy gave a satisfactory result in 92% of cases. In the majority of cases where tumour was disagnosed the specimen was of sufficient size to make a definite histological diagnosis even without a clinical history.

In 20% of cases history was necessary to diagnose the histological type of tumour and in 4% of cases, either because of the small size of the specimen or the type of biopsy, history was necessary to differentiate tumour from benign tissue. As was to be expected diagnosis was more difficult in the reticuloses and sarcomata. In our opinion this method of drill biopsy gives satisfactory sections more frequently with less distortion than more conventional needle biopsy methods.

### SUMMARY

This article describes the results obtained in 500 biopsies using a pneumatic drill biopsy machine. An assessment has been made of the adequacy of the amount of tissue obtained. In the majority of biopsies it was possible to make a firm diagnosis on the tissue, but in 20% of cases it was necessary to refer to the clinical history to determine the type of malignancy. It is thought that the biopsy was correct in at least 92% of the cases.

### REFERENCES

Morrison, R. and Deeley, T. J.—(1955) *J. Fac. Radiol.*, **6**, 287.
Deeley, T. J.—(1960) *Acta Un. int. Cancr.*, **16**, 338.